# Intermittent hypoxia inhibits mandibular cartilage growth with reduced TGF-β and SOX9 expressions in neonatal rats

**DOI:** 10.1038/s41598-020-80303-3

**Published:** 2021-01-13

**Authors:** Kochakorn Lekvijittada, Jun Hosomichi, Hideyuki Maeda, Haixin Hong, Chidsanu Changsiripun, Yo-ichiro Kuma, Shuji Oishi, Jun-ichi Suzuki, Ken-ichi Yoshida, Takashi Ono

**Affiliations:** 1 Department of Orthodontic Science, Graduate School of Medical and Dental Sciences, Tokyo Medical and Dental University (TMDU), 1-5-45 Yushima, Bunkyo-ku, Tokyo, 113-8549 Japan; 2 Department of Orthodontics, Faculty of Dentistry, Chulalongkorn University, Bangkok, Thailand; 3 Department of Forensic Medicine, Graduate School of Medicine, Tokyo Medical University, Tokyo, Japan; 4 Department of Advanced Clinical Science and Therapeutics, Graduate School of Medicine, The University of Tokyo, Tokyo, Japan

**Keywords:** Cartilage development, Disease model, Bone development, Physiology, Respiration, Experimental models of disease, Medical research, Translational research

## Abstract

Intermittent hypoxia (IH) has been associated with skeletal growth. However, the influence of IH on cartilage growth and metabolism is unknown. We compared the effects of IH on chondrocyte proliferation and maturation in the mandibular condyle fibrocartilage and tibial hyaline cartilage of 1-week-old male Sprague–Dawley rats. The rats were exposed to normoxic air (n = 9) or IH at 20 cycles/h (nadir, 4% O_2_; peak, 21% O_2_; 0% CO_2_) (n = 9) for 8 h each day. IH impeded body weight gain, but not tibial elongation. IH also increased cancellous bone mineral and volumetric bone mineral densities in the mandibular condylar head. The mandibular condylar became thinner, but the tibial cartilage did not. IH reduced maturative and increased hypertrophic chondrocytic layers of the middle and posterior mandibular cartilage. PCR showed that IH shifted proliferation and maturation in mandibular condyle fibrocartilage toward hypertrophic differentiation and ossification by downregulating TGF-β and SOX9, and upregulating collagen X. These effects were absent in the tibial growth plate hyaline cartilage. Our results showed that neonatal rats exposed to IH displayed underdeveloped mandibular ramus/condyles, while suppression of chondrogenesis marker expression was detected in the growth-restricted condylar cartilage.

## Introduction

Intermittent hypoxia (IH) is one of the preceding symptoms of apnea of prematurity (AOP) and sudden infant death syndrome (SIDS)^1,2^. Moreover, IH can cause neurobehavioral impairment and developmental retardation^3–6^. Clinical studies have provided evidence that adenotonsillectomy and continuous positive airway pressure may prevent orofacial growth retardation^7,8^. Prepubescent rodents subjected to IH exhibited craniofacial and mandibular growth retardation, nasal bone hypoplasia, reduced nasal cavity volume^9^, elevated bone density^10,11^, and underdeveloped mandibular ramus/condyle units^12^. Craniofacial growth is fastest during the first 3 to 5 years in humans^13^ and during the first 3 weeks in rodents^14^. Whether IH alters chondrocyte metabolism despite rapid skeletal growth at the cartilage is unknown.

The temporomandibular joint (TMJ) contains fibrocartilage derived from embryonic secondary chondrocytes. Mandibular growth and remodeling occurs in the epiphyseal cartilage of the mandibular condylar head^15^. TMJ epiphyseal cartilage, hyaline cartilage, and primary cartilaginous joints differ developmentally from limb growth plates in terms of cell composition and organization, extracellular matrix components, and embryonic origins^16^. Fibrocartilage and hyaline cartilage respond differently to transforming growth factor-beta (TGF-β)^17,18^. TMJ and limb chondrocytes express type II and X collagen, SRY-Box transcription factor 9 (SOX9), and cartilage oligomeric matrix protein (COMP). Collagen type X is upregulated in hypertrophic chondrocytes^19,20^ and reflects endochondral ossification^21^. SOX9 induces TGF-β/Smad signaling, which triggers chondrogenic differentiation, upregulates type II collagen, and downregulates type X collagen in chondrocytes^22,23^. SOX9 biases RUNX2 and β-catenin signaling toward endochondral osteogenesis and impedes hypertrophic chondrocyte conversion to osteoblasts and osteocytes^47^.

IH has a stronger influence on the longitudinal growth of the rat mandible than on the tibia^9–11^. Cartilage and bone metabolism in the fibrous cartilage of the TMJ may be affected by IH differently from that in the hyaline cartilage of the limb growth plate. Therefore, we aimed to compare the response of cartilage growth in the mandibular condyl (fibrocartilage) and the tibial growth plate (hyaline cartilage) to IH, and to thus investigate temporal chondrogenesis marker expression. Our subsequent findings provide a clearer picture of the response of condylar chondrocytes to IH in a neonatal in vivo model.

## Results

### Systemic growth of neonatal rats subjected to IH

Body weight and tibial length were recorded as indices of systemic growth. One week after IH induction, the body weight of the IH group was significantly lower than that of the normoxic (N) group (Table 1). However, tibial length did not differ between the two groups (Table 2). These findings were consistent with those of previous reports^9–11^.

**Table 1:** Changes in body weight (g).

Day	N group (n = 9)		IH group (n = 9)		*P-*value
	Mean	SD	Mean	SD	
0	14.09	0.63	13.33	1.45	0.201
7	30.02	4.86	20.5	2.82	*

**Table 2:** Comparison of tibial length (mm) between the N and IH groups at day 7.

N group (n = 9)		IH group (n = 9)		*P-*value
Mean	SD	Mean	SD	
8.86	0.44	8.48	0.7	0.175

### Differential growth of craniofacial bones during IH

**Figure 1:**
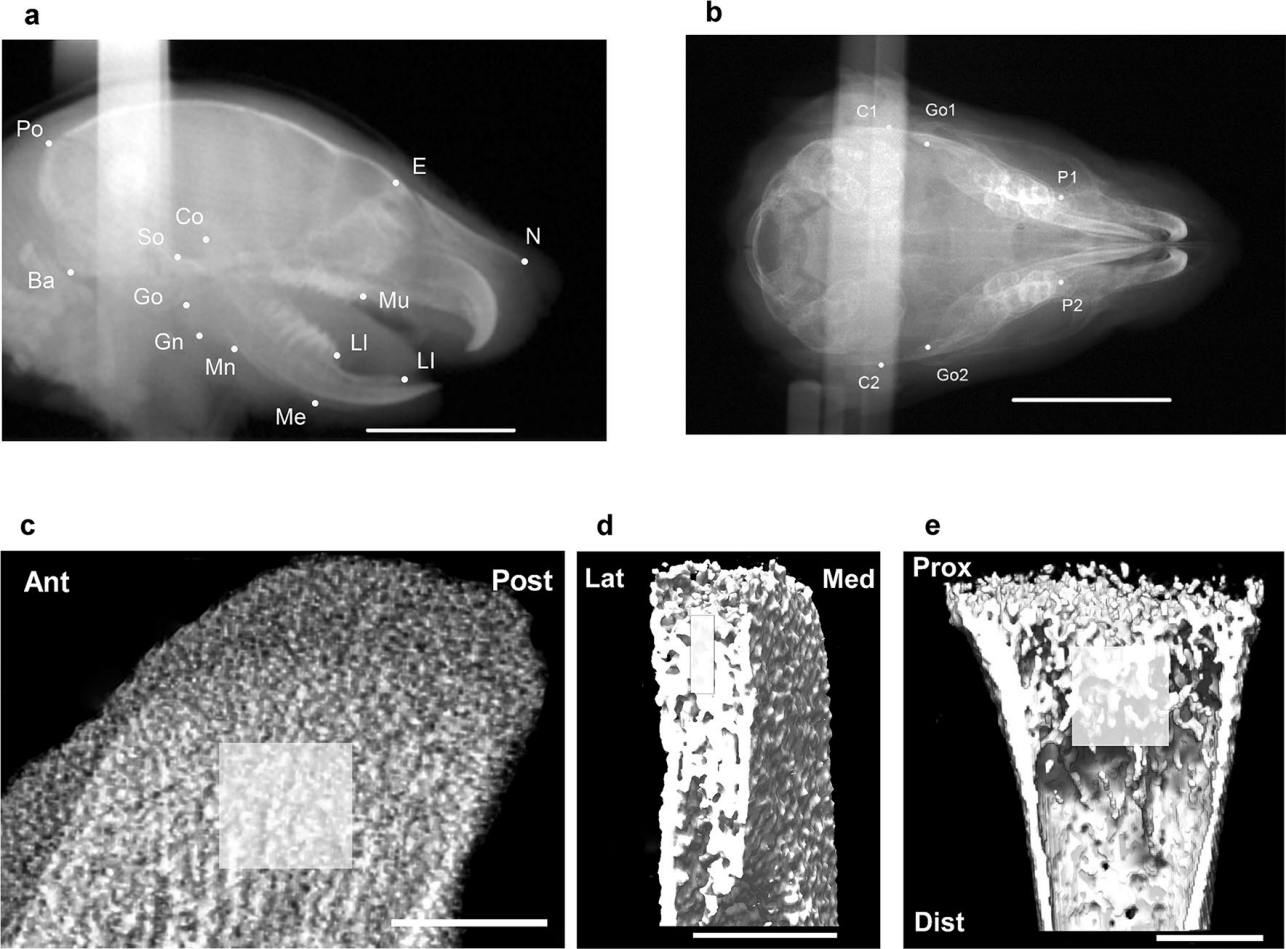
Cephalometric and μCT analysis landmarks. (**a**) Lateral cephalometric and (**b**) dorsoventral cephalometric view in a 2-week-old rat. Region of interest (ROI) in μCT images, including sagittal (**c**) and coronal (**d**) views of mandibular condyle and coronal (**e**) view of proximal tibial epiphysis. Boxes in (**c**–**e**) indicate the ROI. Scale bar = 10 mm (**a**,**b**) and 50 μm (**c**–**e**). *Ant* anterior, *Dist* distal, *Lat* lateral, *Med* medial, *Post* posterior, *Prox* proximal.

**Figure 2:**
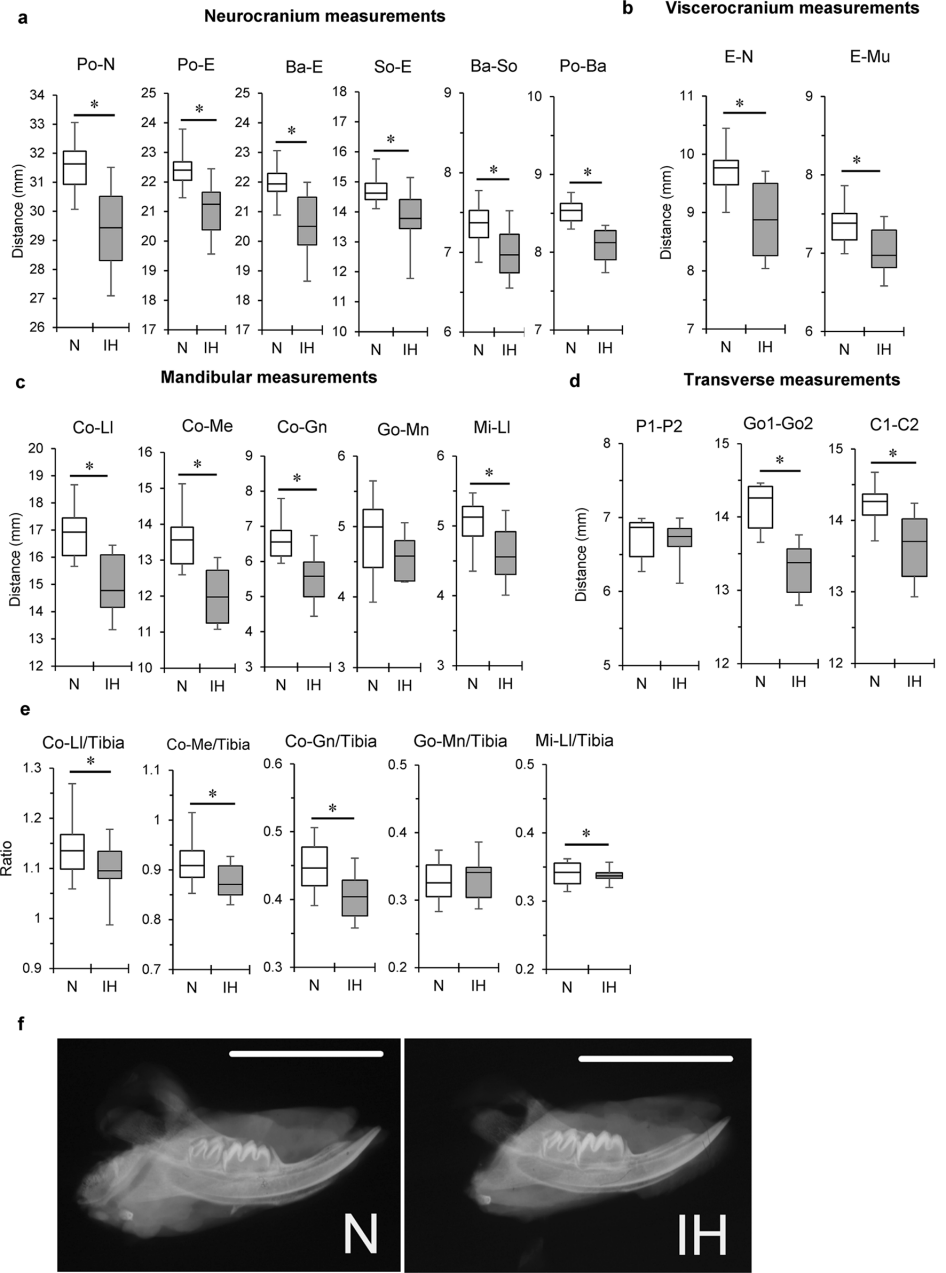
Differential craniofacial growth in infant rats subjected to IH. Linear craniofacial measurements obtained from lateral and transverse cephalometric images of 2-week-old rats. Neurocranial (**a**), viscerocranial (**b**), mandibular (**c**), and transverse (**d**) parameters of normoxic (N) and IH group cephalometry. (**e**) Normalization of mandibular dimensions to tibial length in N and IH rats. Ratios of mandibular length (Co-Ll, Co-Me), ramus height (Co-Gn), and anterior corpus length (Mi-Ll) to tibial length were significantly smaller in the IH group than those in the N group. (**f**) Representative lateral radiographs of hemi-mandible from infant rat after one week of IH exposure. The abbreviations are explained in Fig. 2a and Tables 1A and 1B. Scale bar = 10.0 mm. Data are mean ± SEM for each group. *P < 0.05.

All cephalometric measurements, except posterior corpus length (Go-Mn) and palatal width (P1–P2), were significantly lower in the IH group than in the N group (Figs. 1a,b, 2a–d, Supplementary Table S1a,b). Tibial length was not affected by IH. The ratios of the mandibular length (Co-Ll and Co-Me), ramus height (Co-Gn), and anterior corpus length (Mi-Ll) to the tibial length were significantly reduced by IH. The ratios of the neurocranial and viscerocranial parameters to tibial length were not affected by IH (Fig. 2e, Supplementary Table S1a,b). Thus, IH markedly reduced mandible size, which includes the condyle-ramus region (Fig. 2f).

### Effect of IH on bone mineralization in the mandibular condyle and tibial cartilage in neonatal rats

**Figure 3:**
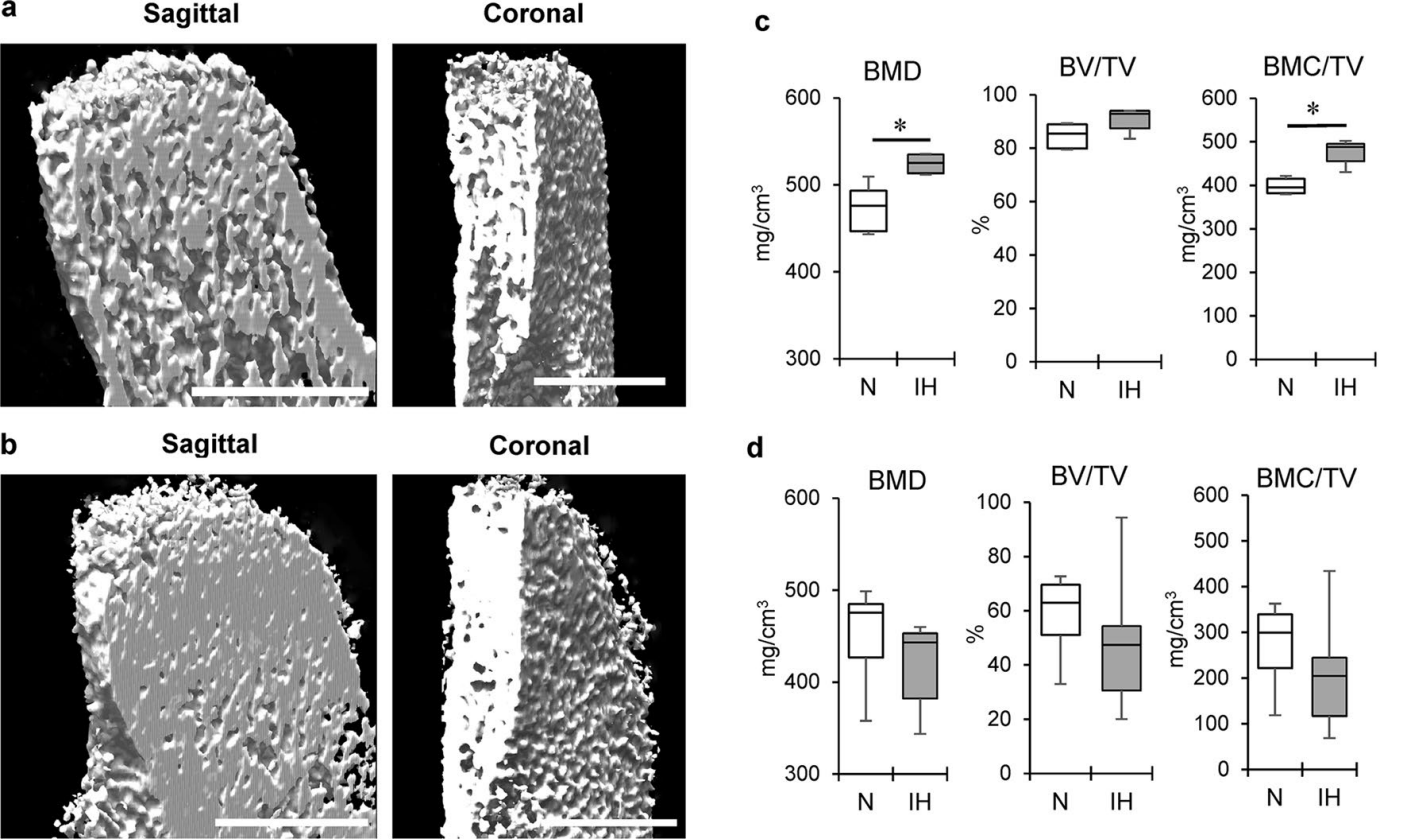
IH-induced high bone mineral density in mandibular condylar head in contrast to tibial epiphysis. Representative sagittal and coronal sections of mandibular condyle in the N (**a**) and IH (**b**) groups on 3D μCT imaging. Panel (**c**) shows comparative quantification of bone microstructural parameters in mandibular condyle cancelous bone between N and IH groups. Panel (**d**) shows comparisons of cancelous bone of proximal tibial epiphysis in both groups. BMD, bone mineral density; BV/TV, bone volume/trabecular volume; BMC/TV, bone mineral content/trabecular volume. Scale bar = 50 μm. Data are mean ± SEM for each group. *P < 0.05.

Bone mineralization was evaluated using three-dimensional micro-computed tomography (3D μCT) analysis. IH significantly increased cancellous bone density in the mandible (Figs. 1c,d, 3a–c), but not in the tibia (Figs. 1e, 3d). Quantification of the bone microstructural parameters confirmed that IH increased both areal bone mineral density (BMD) and bone mineral content/volumetric bone mineral density (BMC/TV) in the cancellous bone of the mandibular condyle, but not the tibia.

### Histological changes in the cartilaginous layers of the mandibular condyle and tibial epiphysis

#### Mandibular condyle

**Figure 4:**
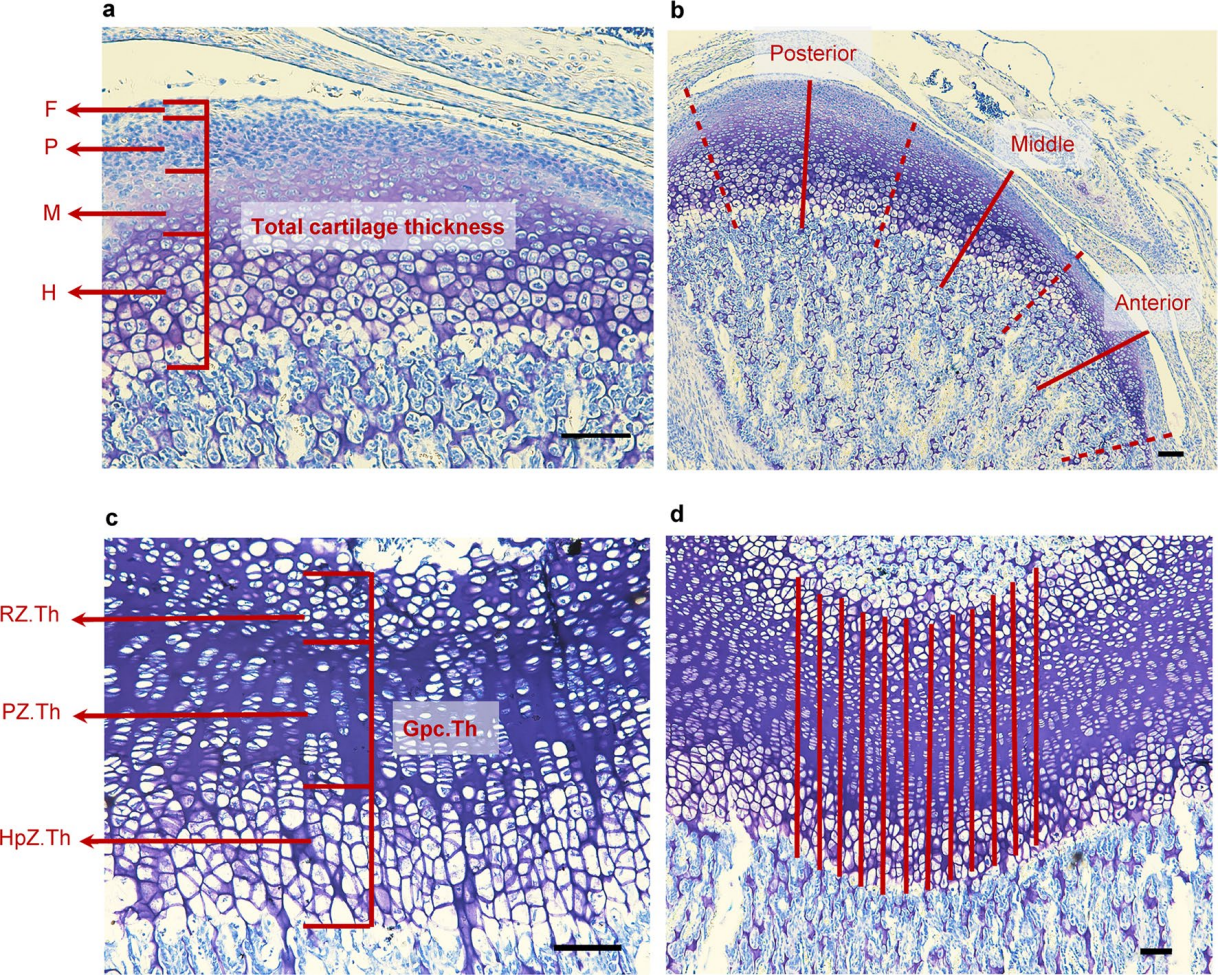
Histological images of mandibular condylar cartilage and tibial growth plate from a 2-week-old rat following toluidine blue staining. (**a**) Sagittal section of mandibular condylar cartilage. Black dashed lines show the divided region of condyle, including the fibrous layer (F), proliferating layer (P), maturative layer (M), and hypertrophic layer (H). Red lines in (**b**) show the center of each part used to measure thickness of mandibular cartilage. (**c**) Longitudinal section of tibial cartilage, including the resting zone (RZ.Th), proliferative zone (PZ.Th), hypertrophic zone (HpZ.Th), and height of growth plate (Gpc.Th). (**d**) Twelve parallel lines drawn through tibial cartilage to permit measurements. Scale bar = 100 μm.

**Figure 5:**
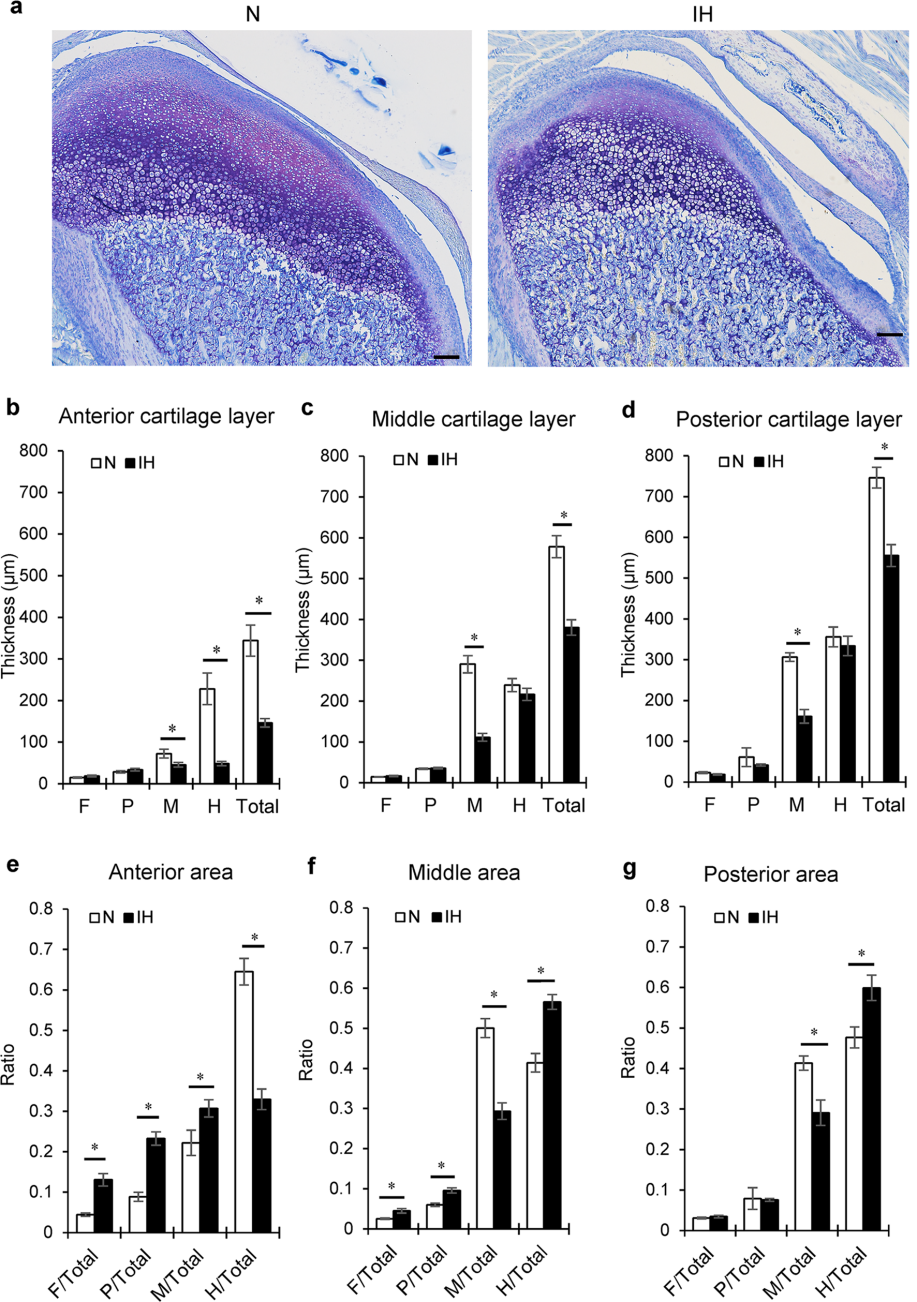
Histological changes in mandibular condylar cartilage of IH rats. (**a**) Representative images of toluidine blue-stained sagittal sections of mandibular condyle from the N and IH groups. Thickness of each cartilage layer of anterior (**b**), middle (**c**), and posterior (**d**) parts in mandibular condyle. Ratios of each cartilage layer to total cartilage thickness shown in panels (**e**–**g**). *F* fibrous layer, *P* proliferating layer, *M* maturative layer, *H* hypertrophic layer. Scale bar = 100 μm. Data are mean ± SEM for each group. *P < 0.05.

The thicknesses of the total cartilaginous layers in the anterior, middle, and posterior areas of the condylar cartilage dramatically decreased during IH (Figs. 4a,b, 5a). After 1 week, the thickness of the maturative layer was largest in the middle of the N group of rats. In contrast, IH significantly reduced the maturative layer in all areas (Fig. 5b–d). IH substantially reduced the hypertrophic layer in the anterior area. However, the hypertrophic layers in the middle and posterior areas were comparable in the N and IH groups (Fig. 5b–d).

The relative thickness of each layer was then calculated to determine whether IH exposure influenced chondrocyte maturation in each layer. After 1 week of IH exposure, the relative thickness of the maturative layer was significantly reduced in the middle and posterior areas, but increased in the anterior area (Fig. 5e–g). In contrast, IH markedly decreased the hypertrophic layer in the anterior area but significantly increased the maturative layer relative to the total cartilage (Fig. 5e). IH significantly reduced the maturative layer in the middle and posterior areas, but increased the hypertrophic layer relative to the total cartilage (Fig. 5f–g).

#### Tibial epiphysis

**Figure 6:**
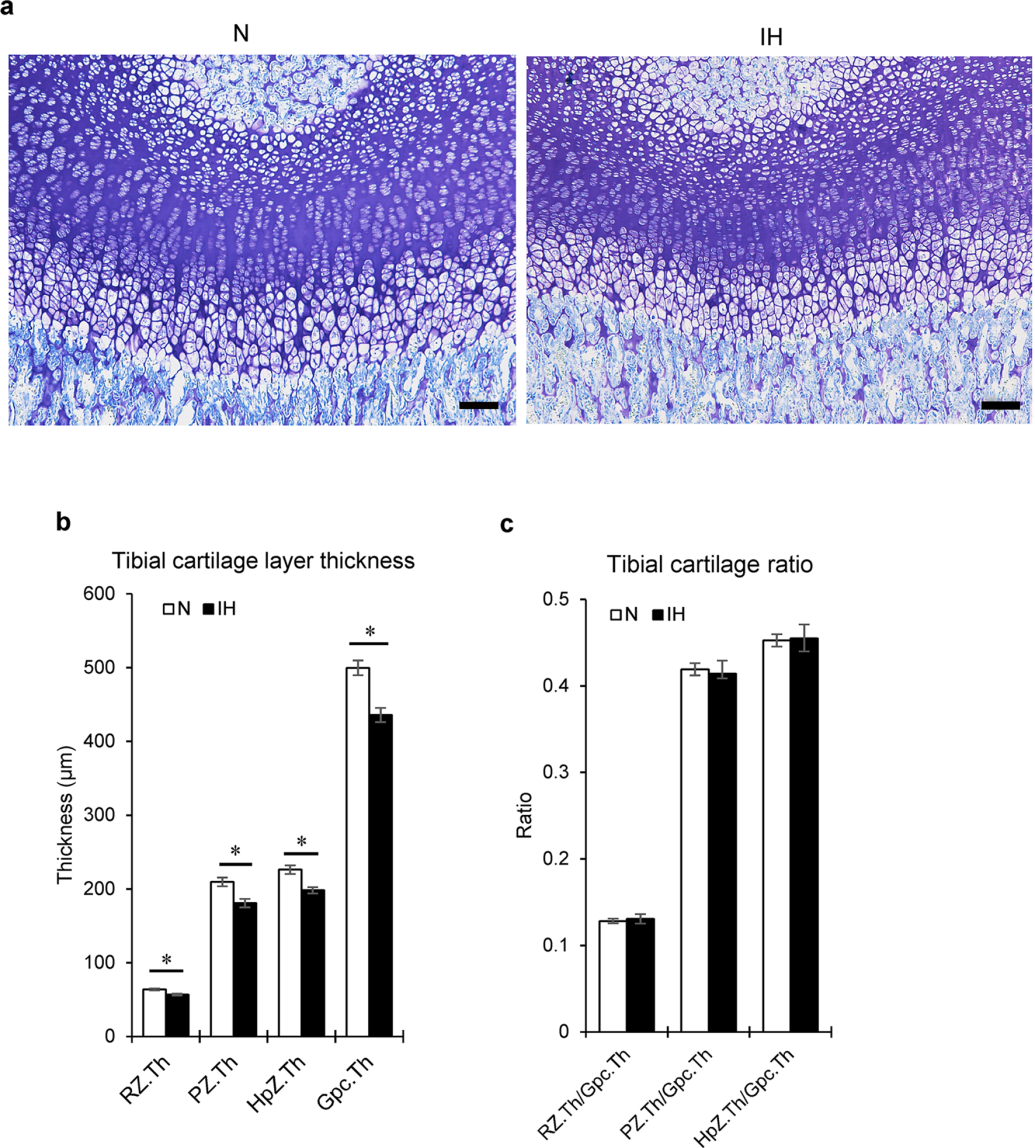
Histological changes in tibial cartilage of IH rats. (**a**) Representative images of toluidine blue-stained longitudinal sections of tibial cartilages from the N and IH groups. (**b**) Comparison of thicknesses of cartilage layers and growth plates between the N and IH groups. (**c**) Ratios of cartilage layer thickness to growth plate thickness. *RZ.Th* tibial cartilage layers, *RZ.Th* resting zone, *PZ.Th* proliferative zone, *HpZ.Th* hypertrophic zone, *Gpc.Th* height of growth plate. Scale bar = 100 μm. Data are mean ± SEM for each group. *P < 0.05.

IH reduced the thicknesses of all cartilage layers, including the growth plate in the proximal tibial epiphysis (Gpc.Th) (Figs. 4c,d, 6a,b). However, the relative thickness of each layer did not significantly differ from that of the total cartilage in the tibia (Fig. 6c).

### Differential gene expression is associated with chondrocyte metabolism in the mandibular condyle and tibia during IH

**Figure 7:**
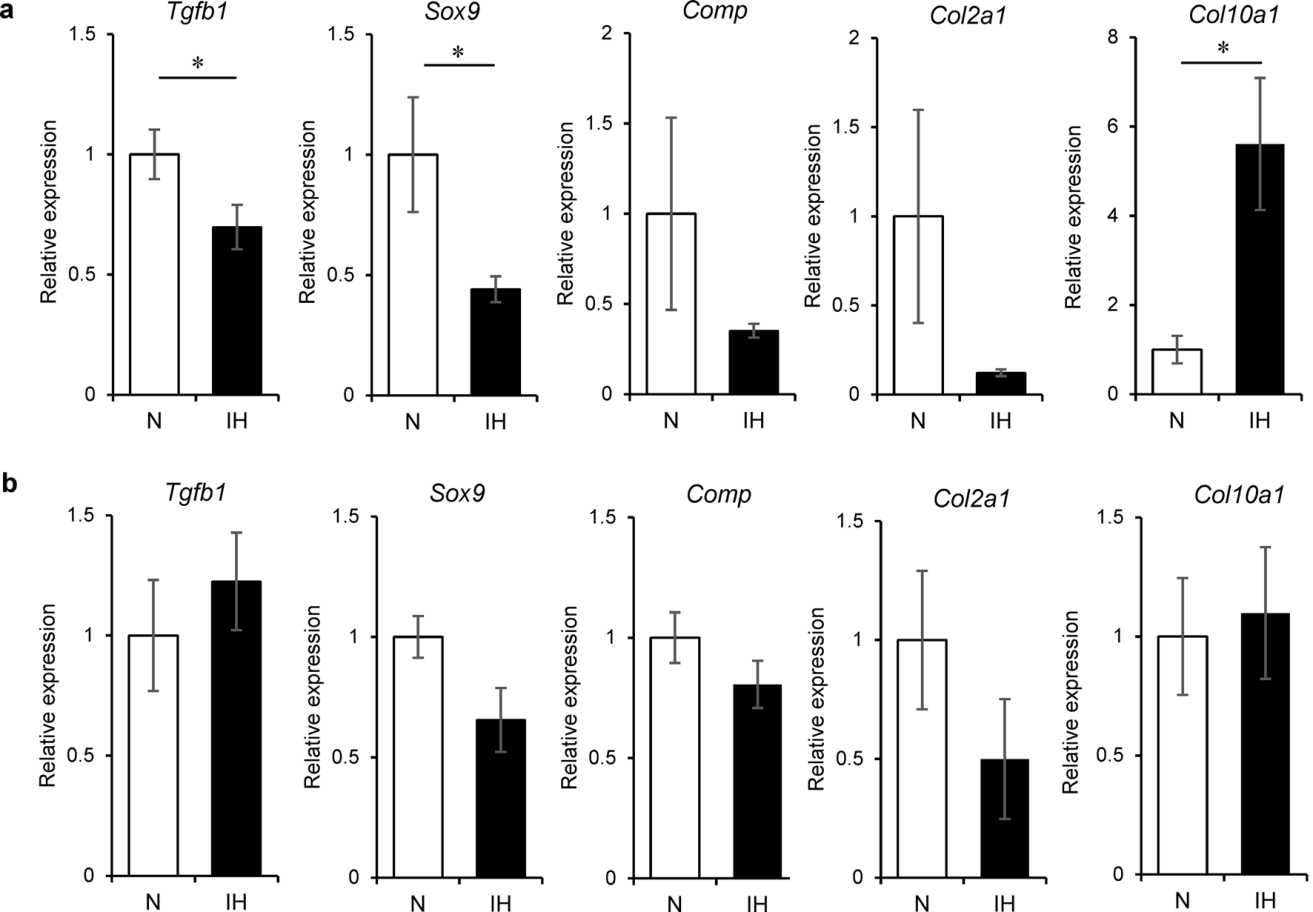
Differential effects of IH on expression levels of genes associated with chondrocyte and bone metabolism in mandibular condyle and tibia. Relative mRNA expression levels of bone metabolic regulator TGF-β (*Tgfb1*) and chondrocyte markers SOX9 (*Sox9*)*,* COMP (*Comp*), Collagen II (*Col2a1*), Collagen X (*Col10a1*), and RUNX2 (*Runx2*) in mandibular condylar cartilage (**a**) and tibial cartilage (**b**). Data are mean ± SEM for each group. *P < 0.05.

IH modified the chondrocyte phenotype in the mandibular condyle. The genes related to chondrogenesis were examined in the mandibular condylar cartilage and tibial epiphyseal plate. In the former, IH significantly reduced the mRNA levels of the chondrogenesis markers TGF-β (*Tgfb1*) and SOX9 (*Sox9*) (Fig. 7a). It also increased the mRNA level of collagen X (*Col10a1*), a marker of hypertrophic chondrocytes, but not that of collagen II (*Col2a1*) mRNA (a marker of early chondrocyte differentiation in the mandibular condyle). RUNX2 (*Runx2*) mRNA expression levels were comparable between the IH and N rats. In contrast, IH did not affect the expression levels of these genes in the tibial cartilage (Fig. 7b). In neonatal mandibular condylar cartilage, IH-dependent chondrocyte hypertrophy and suppression of chondrogenesis occur via the TGF-β/SOX9 pathway.

## Discussion

To the best of our knowledge, this study is the first to demonstrate that IH exposure impairs mandibular fibrocartilage growth and induces chondrocyte hypertrophy and endochondral ossification in neonatal rats. In contrast, IH did not induce these effects in the tibial growth plate (hyaline) cartilage. One week of IH exposure repressed the chondrogenesis regulators TGF-β (*Tgfb1*) and SOX9 (*Sox9*) in the fibrocartilage of the rodent mandible condyle. The mRNA of collagen X (*Col10a1*), a hypertrophic chondrocyte marker, was upregulated in the mandibular condyle of neonatal rats. IH induced BMD and BMC/TV in the mandibular condyle, thereby promoting chondrocyte hypertrophy and cartilage calcification. In contrast, IH had no such effects on hyaline cartilage or tibial growth plate bone. Therefore, IH impedes mandible growth in neonatal rats, and mandibular condylar fibrocartilage is sensitive to IH.

Since the rat is the most commonly used model in numerous animal studies, the differences in anatomy, physiology, and development between different species must be considered. Information on the correlation between animal and human age is necessary. However, the method of translating the time points in rats into human developmental stages is not completely clear. Hence, our findings should be interpreted with caution^25^.

Lateral and dorsoventral cephalometric radiographs revealed relatively reduced mandibular length (Co-Ll and Co-Me), ramus height (Co-Gn), and bigonial width (Go1-Go2) in neonatal rats exposed to IH. These findings are similar to those reported for the mandibular morphology of pubescent rats subjected to IH^11,12^. However, no reduction in the posterior corpus length (Go-Mn) was observed in neonatal rats exposed to IH. Human and rodent mandibles consist mainly of a horizontal portion (mandibular body) and two perpendicular portions (mandibular ramus). Bone growth in the mandibular body and ramus is complex^13,14^. The posterior corpus (Go-Mn) is the main site of masseter muscular attachment in the rat mandible. Masticatory muscle development strongly influences bone formation^26^. Masseter muscle development begins in rats at the weaning stage (3 weeks) when the animals begin biting and chewing solid food^27^. We measured the ramus portion, where the mandibular condyle controls skeletal growth^28^. In rodents, bone growth is very rapid during the first 3 weeks of life^29^. In our 1–2-week neonatal rat models, mandibular underdevelopment was explained by the IH-induced changes in condylar cartilaginous growth, rather than by masticatory muscle development.

The μCT data revealed dense epiphyseal bone in the mandibular condylar heads of neonatal rats exposed to IH. The IH treatment significantly upregulated BMD and BMC/TV in the cancellous bone region of the mandibular condyle. The bony microstructural changes occurring in adult patients with obstructive sleep apnea (OSA) are controversial^30^. Positive BMD responses were reported for the alveolar bone of adolescent rats^10^ and the lumbar vertebrae of adult rats^31^ exposed to IH. However, a neonatal rat model exposed to IH for 1 week presented with reduced BMD in the mandible^32^. Relative to the control, IH exposure did not significantly modify BMD in adult mice^33^. To the best of our knowledge, no reports have clarified the roles of hypoxia in cartilage and bone metabolism in IH-exposed neonates.

Rapid calcified matrix removal and marrow cavity formation are essential for growth plate development during endochondral bone biosynthesis^29^. Usui and colleagues suggested that hypertrophic chondrocytes regulate osteoclastogenesis in the growth plate and remove calcified matrix^34^. This process is mediated by BMP2-induced RANKL expression in normal bone. Here, the markedly increased BMD observed in the mandibular condyles of neonatal rats exposed to IH was not accompanied by any change in the Runx2 mRNA level. The latter has been associated with bone metabolism. The mechanisms underlying the effect of IH on cartilage and bone metabolism in neonatal rats remain to be determined. Hypoxia and osteogenesis are related via activation of the hypoxia-inducible factor (HIF) pathway, which occurs when the oxygen level is low, leading to marked changes in bone density together with increased vascular endothelial growth factor-A (VEGF-A) levels^35^. A previous in vitro study reported increased expression of HIF-1α and VEGF in condylar chondrocytes under chemically induced hypoxia^36^. HIF-1α has protective and anti-apoptotic effects on chondrocytes during skeletogenesis. A study on HIF-1α gene-knockout mice reported varied skeletal growth through HIF1α-regulated chondrogenesis^37^. Hence, we also performed RT-PCR for HIF-1α and HIF-2α gene expression in the mandibular condylar cartilage and tibial cartilage of neonatal rats exposed to IH. No significant difference in HIF-1α expression was evident in condylar chondrocytes (Supplementary Fig. S1). Consistent with our results, Bianchi and colleagues reported age-related HIF-1α expression after IH exposure. The neonatal rats did not show any changes in HIF-1α expression. The authors suggested that this might be due to the adaptation ability of neonates^38^. Based on these findings, bone morphogenetic protein 2-induced RANKL and HIFs, and their downstream gene expression should be evaluated in future studies. In addition to qRT-PCR, western blot analysis for protein detection is necessary to confirm these findings. Furthermore, the previous biomarker study demonstrated excessive reactive oxygen species (ROS) generation in rodents exposed to IH^6^. Oxidative stress arrests chondrocyte proliferation and degrades cartilage in the growth plate^19^. Accordingly, further investigations on the role of ROS in these conditions are warranted. Additionally, small interfering RNA or RNA interference studies may prove useful in revealing the direct causality between chondrocyte metabolism and related gene expression during IH exposure.

Regiospecific changes in the maturative and hypertrophic layers were observed in the mandibular epiphyseal cartilage of neonatal rats exposed to IH for 1 week. The qRT-PCR analysis revealed significantly downregulated TGF-β (*Tgfb1*) and SOX9 (*Sox9*) and upregulated collagen X (*Col10a1*) mRNA, which is relevant to our histomorphology appearance, in the mandibular condylar cartilage of the IH group compared to that in the N group. SOX9 inhibits chondrocyte maturation from the proliferation to prehypertrophy stages and the development of an osteoblastic phenotype^24^. *Sox9*-mutant mice present with dwarfism and their proliferative layers are shortened^24,39^. Advanced endochondral ossification occurs in the growth plate cartilage of the long bones because of premature chondrocyte prehypertrophy and matrix mineralization. *Sox9* knockdown promotes pathological heart valve calcification via RUNX2-mediated osteogenic target gene activation^40^. The regulation of SOX9 expression is complex, although it is known to be involved in various mechanisms at the gene and mRNA levels. Many pathways in chondrogenesis, including the Hedgehog, BMP-2, TGF-β, FGF-2, hypoxia signaling, and inflammatory pathways have been reported to play a role in SOX9 upregulation and downregulation^41–43^. A previous in vitro study reported that Sox9 transduced articular chondrocytes under lowered oxygen conditions (5%) had a better ability to increase cell proliferation and DNA production compared to those in normal conditions^44^. Hence, we speculated that the increase in the hypertrophic layer and endochondral ossification in the mandibular condylar cartilage after IH exposure may be related to a specific mechanism in the SOX9 pathway. However, to support this hypothesis, further experiments on SOX9 overexpression and condylar chondrocyte morphology under IH should be conducted in vivo. Moreover, to provide a better demonstration of the changes in condylar chondrocytes after IH exposure, performing the time-line experimental design or dynamic histomorphology to present the chondrocyte growth pattern at different time points might help clarify these findings.

In conclusion, the present study demonstrates that IH contributes to mandibular condyle growth impediments and morphometric deficits in condylar chondrocytes of neonatal rats, and also alters the expression of chondrogenic regulators. These findings provide a better understanding of the condylar cartilage tissue responses to IH.

## Methods

### Experimental model

The protocol was approved by the Institutional Animal Care and Use Committee of Tokyo Medical University, Japan (Approval No. H29-0021 and H30-0022). All experiments complied with relevant guidelines and regulations. Eighteen 5-day-old male Sprague–Dawley rats and their mothers were housed in computer-controlled environmental chambers. The oxygen concentration was monitored and readjusted every day to maintain normoxic and hypoxic conditions^45^. Seven-day-old pups and their mothers in the IH group were subjected to IH at 20 cycles/h (nadir, 4% O_2_; peak, 21% O_2_; 0% CO_2_) for 8 h each day during the light-on period (12–14 h). The pups and mothers in the N group were exposed to room air throughout the experiment. Animals were maintained under a 12-h light–dark cycle (9:00 AM—5:00 PM) at 24 ± 0.2 °C with ad libitum access to standard chow and water. All pups were euthanized by isoflurane inhalation at 2-weeks-of-age.

### Cephalometric analysis

Lateral and dorsoventral cephalometric radiographs were obtained using a SOFTEX CMB-2 soft X-ray machine (Softex Co. Ltd., Tokyo, Japan) to evaluate dimensional changes in the mandible. Two ear rods fixed the head of each rat in a standard head posture^11,46^. X-ray images of the tibia were taken and used to assess skeletal growth.

### 3D-μCT

Changes in mineralized cartilage and subchondral bone microstructure were investigated by μCT coupled to a desktop X-ray μCT system (Inspexio, Shimadzu, Japan). The output settings were 75 kV and 140 mA^47^. Samples were scanned at a resolution of 20 μm^10,47^. A 1.0 mm × 1.0 mm × 0.2 mm cancelous bone area 1.0 mm from the condylar epiphyseal cartilage was the region of interest (Fig. 1c,d). An area of equal size in the diaphysis 50 μm inferior to the tibial cartilage growth plate was used for bone density analysis (Fig. 1e)^48^. Bone volume/trabecular volume (BV/TV, %), BMD (mg/cm^2^), BMC (mg), and BMC/TV (mg/cm^3^) were calculated. The results were analyzed using TRI/3D-BON software (Ratoc, Tokyo, Japan).

### Histomorphometric analysis

The TMJ, tibia, and surrounding tissues were dissected from the right side, fixed in 4% (v/v) paraformaldehyde for 24 h, decalcified with 4.13% (w/v) EDTA (pH 7.4) at 4 °C for 6 weeks, and embedded in paraffin. Blocks containing the mandibular condyle were cut into 5 μm thick sections parallel to the sagittal plane. The tibiae were cut longitudinally into 5 μm thick sections. The sections were stained with toluidine blue to measure the cartilage thickness.

The mandibular condyle cartilage was divided into fibrous, proliferative, maturative, and hypertrophic cell layers according to cell shape, size, and staining intensity (Fig. 4a)^49^. Condylar cartilage thickness was measured at the anterior, middle, and posterior parts of the central sagittal sections of the mandibular condylar head (Fig. 4b). Three lines were drawn through the middle of each part, starting from the superior border of the fibrous layer to the inferior border of the hypertrophic cell layer (Fig. 4b)^50^. The thickness of each layer and the total cartilage thickness were measured by one observer along the midline of the three portions. The average of six sections was calculated for each animal.

To measure the tibial growth plate cartilage, 12 parallel lines separated by 225 μm were drawn through the cartilage (Fig. 4c)^51^. Twelve thickness measurements were averaged for each section. Six sections per tibia were measured, and the average was calculated. The total height of the growth plate (Gpc.Th) and the thicknesses of the resting (RZ.Th), proliferative (PZ.Th), and hypertrophic (HpZ.Th) zones were measured as previously described (Fig. 4d)^52^. Measurements were made with NIS-Elements Analysis D v. 3.2 (Nikon, Tokyo, Japan).

### qRT-PCR

Total RNA was isolated from the left mandibular condyle and tibia using a PureLink FFPE total RNA isolation kit (Invitrogen, Carlsbad, CA, USA) according to the manufacturer’s protocol. First-strand cDNA was synthesized with a PrimeScript RT reagent kit (TaKaRa Bio, Shiga, Japan). qRT-PCR assays were performed in triplicate for each sample using Premix Ex Taq (TaKaRa Bio) in a StepOnePlus Real-Time PCR System (Applied Biosystems, Foster City, CA, USA). Real-time PCR analyses were conducted using TaqMan gene expression assays (Applied Biosystems) and commercially obtained rat mRNA probes/primers for SOX9 (*Sox9*; Rn01751070_m1), type II collagen (*Col2a1*; Rn01637087_m1), type X collagen (*Col10a1*; Rn01408030_m1), COMP (*Comp*; Rn00563255_m1), Runx2 (*Runx2*; Rn01512298_m1), and hypoxanthine phosphoribosyltransferase 1 (HPRT-1) (*Hprt1*; Rn01527840_m1). *Hprt1* expression served as an internal control. Gene expression levels were calculated using the 2^−ΔΔCT^ relative quantitation method.

### Statistical analyses

Data are presented as the mean ± standard error of the mean. Statistical calculations were performed using IBM SPSS Statistics v. 24 (IBM Corp., Armonk, NY, USA). Data normality and variance were evaluated using *F*-tests. Differences between the IH and N groups in terms of mean weight, tibial length, craniofacial dimensions, bone microstructure, cartilage thickness, and qRT-PCR were evaluated by independent-sample *t*-tests. Statistical significance was set at *P* < 0.05.

## Supplementary Information

Supplementary Information.

## Data Availability

The datasets generated and/or analyzed during the current study are available from the corresponding author upon reasonable request.
