# Supplementary material for: Intermittent hypoxia inhibits mandibular cartilage growth with reduced TGF-β and SOX9 expressions in neonatal rats
Source: Sci Rep. 2021 Jan 13;11:1140. doi: 10.1038/s41598-020-80303-3 (PMC7806651; doi:10.1038/s41598-020-80303-3)
Supplement: Supplementary file 1 — Supplementary Information. [file 41598_2020_80303_MOESM1_ESM.pdf]

**Supplementary information**

# Intermittent Hypoxia Inhibits Mandibular Cartilage Growth with Reduced TGF-β and SOX9 Expressions in Neonatal Rats

**Kochakorn Lekvijittada[1,2,3], Jun Hosomichi[1,3], Hideyuki Maeda[3], Haixin Hong[1,3], Chidsanu Changsiripun[2], Yo-ichiro Kuma[1], Shuji Oishi[1], Jun-ichi Suzuki[4], Ken-ichi Yoshida[3], Takashi Ono[1]**

[1]Department of Orthodontic Science, Graduate School of Medical and Dental Sciences, Tokyo Medical and Dental University (TMDU), Tokyo, Japan

[2]Department of Orthodontics, Faculty of Dentistry, Chulalongkorn University, Bangkok, Thailand

[3]Department of Forensic Medicine, Graduate School of Medicine, Tokyo Medical University, Tokyo, Japan

[4]Department of Advanced Clinical Science and Therapeutics, Graduate School of Medicine, The University of Tokyo, Tokyo, Japan

**Supplementary Table S1a. Definitions of landmarks in radiographic images**

| | | |
|---|---|---|
| Lateral view landmarks | N | The most anterior point on the nasal bone |
| | E | The intersection of the frontal bone and floor of anterior cranial fossa |
| | Po | The most posterior and superior points on the skull |
| | Co | The most posterior and superior points on the mandibular condyle |
| | So | The intersection of the most anterior tympanic bulla and the superior border of the sphenoid corpus |
| | Ba | The most posterior and inferior points on the occipital condyle |
| | Go | The most posterior point on the mandibular ramus |
| | Gn | The most inferior point on the ramus that lies on a perpendicular bisector of the line Go-Mn |
| | Mn | The most concave portion of the concavity on the inferior border of the mandibular corpus |
| | Me | The most inferior and anterior points of the lower border of the mandible |
| | Mu | The junction of the alveolar bone and the mesial surface of the first maxillary molar |
| | Mi | The junction of the alveolar bone and the mesial surface of the first mandibular molar |
| | LI | The most anterior and superior points on the alveolar bone of the mandibular incisor |
| Dorsoventral view landmarks | Go1, Go2 | The points on the angle of the mandible that produce the widest width; Go1 is the point on the left and Go2 is the point on the right |
| | C1, C2 | The points on the cranium that produce the widest cranial width; C1 is the point on the left and C2 is the point on the right |
| | P1, P2 | The most anterior and medial points within the temporal fossae that produce the narrowest palatal width; P1 is the point on the left and P2 is the point on the right |

**Supplementary Table S1b. Cephalometric measurements**

| | | |
|---|---|---|
| Neurocranium | Po-N | Total skull length |
| | Po-E | Cranial vault length |
| | Ba-E | Total cranial base length |
| | So-E | Anterior cranial base length |
| | Ba-So | Posterior cranial base length |
| | Po-Ba | Posterior neurocranium height |
| Viscerocranium | E-N | Nasal length |
| | E-Mu | Viscerocranium height |
| Mandible | Co-LI | Total mandibular length |
| | Co-Me | Length from condylar head to Me |
| | Co-Gn | Ramus height |
| | Go-Mn | Posterior corpus length |
| | Mi-LI | Anterior corpus length |
| Transverse measurements | Go1-Go2 | Bigonial width |
| | C1-C2 | Maximum cranial width |
| | P1-P2 | Palatal width |

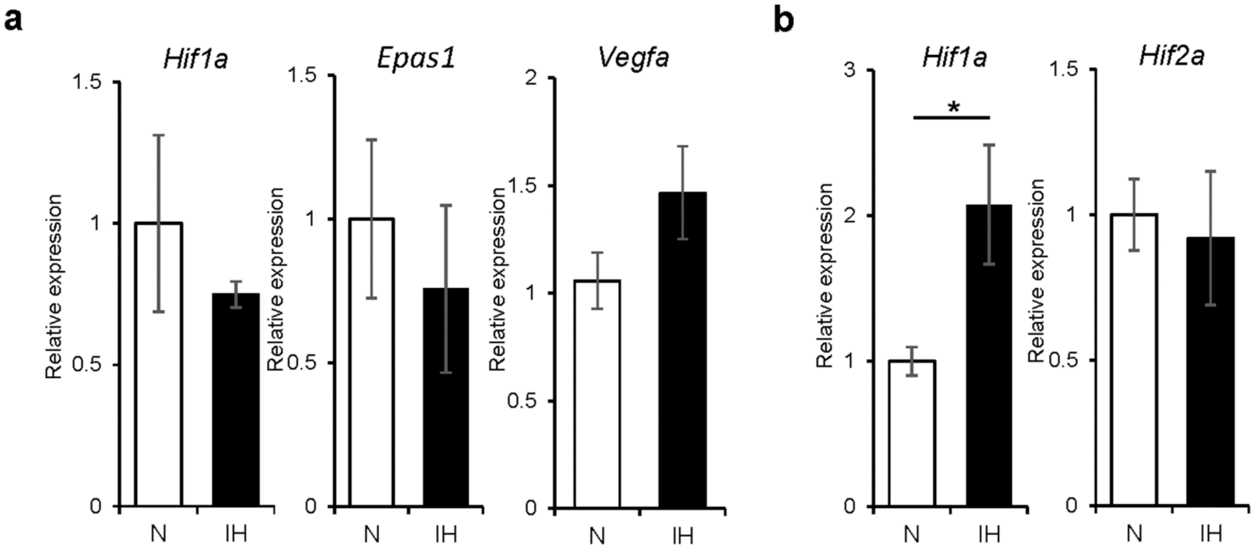


**Supplementary Figure. S1. Gene expression levels of HIFs and VEGF in mandibular condyle and tibia.** Relative gene expression levels of HIF-1α (*Hif1a*), HIF-2α (*Epas1*) and VEGF (*Vefga*) were compared by TaqMan Gene Expression Assays (*Hif1a,* Rn01472831_m1; *Epas1*; Rn00576515_m1; *VEGF;* Rn01511602_m1*; Hprt1* (internalcontrol), Rn01527840_m1). Relative mRNA expression levels of *Hif1a*, *Epas1 and Vegfa* in the mandibular condylar cartilage (a) and relative mRNA expression levels of *Hif1a* and *Epas1 in* the tibial cartilage (b). Data are presented as mean ± SEM for each group. *: $p < 0.05$.
